# The Efficacy of a Combination of Selected Azole Antifungals and Plant Essential Oil Components Against *Malassezia pachydermatis*

**DOI:** 10.3390/jof11040272

**Published:** 2025-04-01

**Authors:** Eva Čonková, Shiri Karasenti, Peter Váczi, Zuzana Malinovská, Miriam Bačkorová

**Affiliations:** 1 Department of Pharmacology and Toxicology, University of Veterinary Medicine and Pharmacy in Košice, Komenského 73, 04181 Košice, Slovakia; peter.vaczi@uvlf.sk (P.V.); zuzana.malinovska@uvlf.sk (Z.M.); 2 Karmiel Veterinary Center, Mivtza Nahshon 4, Karmiel 2198613, Israel; k.shiri89@gmail.com; 3 Department of Pharmaceutical Technology, Pharmacognosy and Botany, University of Veterinary Medicine and Pharmacy in Košice, Komenského 73, 04181 Košice, Slovakia; miriam.backorova@uvlf.sk

**Keywords:** *Malassezia pachydermatis*, azole antifungals, plant essential components, synergistic activity

## Abstract

Infections caused by *Malassezia* (*M.*) *pachydermatis* in dogs are mostly treated with azole antifungals. Excessive use of these drugs is usually associated with an increased incidence of resistant isolates, which can be prevented by combining commonly used antifungals with natural bioactive compounds. The present study aimed at testing the effectiveness of a combination of selected azole derivatives showing low antifungal activity against *M. pachydermatis* isolates, with plant essential oil components displaying the highest efficacy. Among the four azole antifungals tested (itraconazole, posaconazole, clotrimazole, and miconazole), clotrimazole (a mean MIC of 7.62 μg/mL at 72 h and 7.24 μg/mL at 96 h) and miconazole (a mean MIC of 1.71 μg/mL at 72 h and 2.33 μg/mL at 96 h) exhibited the lowest antifungal efficacy. Out of the four plant essential oil components tested (eugenol, terpinene-4-ol, geraniol, and limonene), eugenol (an average MIC of 378.57 μg/mL at 72 h and 1180 μg/mL at 92 h) showed the highest antifungal activity. The checkerboard method was used to assess the interaction of these agents. The fractional inhibitory concentration index (FICI) values for the combination of clotrimazole with eugenol reached 1.43 at 72 h and 0.70 at 96 h and for the combination of miconazole with eugenol, 1.30 at 72 h and 0.45 at 96 h. A higher effect of the combinations was recorded at 96 h, when the combination of clotrimazole with eugenol showed an additive effect in 66.67% of the isolates, and the combination of miconazole and eugenol brought a synergistic effect in 57.14% of the isolates. The obtained results indicate that eugenol is a suitable agent for enhancing the efficacy of poor azoles against *M. pachydermatis*.

## 1. Introduction

*Malassezia pachydermatis*, considered a natural skin and mucosal commensal in dogs, plays an important role in secondary infections, manifesting as otitis or dermatitis in the case of yeast overgrowth [1,2]. The pathogenic potential of *M. pachydermatis* is associated with the activation of yeast virulence factors in combination with the metabolic, hormonal, and immunological status of the animal [3,4]. Poor hygiene, increased humidity and temperature, and the anatomy of the ear allow for favourable growth and yeast multiplication on the skin and auditory canal. *Malassezia* dermatitis is clinically manifested by excessive sebum production and/or a decreased quality of sebum (seborrhoea), the accumulation of moisture, damage of the epidermis and concomitant dermatoses, atopy, and ectoparasitic and bacterial skin infections. Primary diseases involved in the development of infection comprise hypersensitivity (hypersensitivity to fleabite, adverse cutaneous food reactions, and contact allergy); keratinisation disorders; endocrine disease (hyperadrenocorticism, hypothyroidism, and diabetes mellitus); and autoimmune diseases [5,6,7]. *Malassezia* otitis is usually associated with pendulous pinnae, anatomical anomalies or changes that create ear canal stenosis, an increased secretion or retention of cerumen, moisture, and the inhibition of air circulation. Primary causes, such foreign bodies and parasites (especially the ear mite *Otodectes cynotis*), may also be involved in ear inflammation. Suspicion of *Malassezia* otitis occurs when the ears are pruritic, with erythema, and present the accumulation of a brownish discharge [8,9].

Two classes of antifungals, azole and polyenes, are often used to treat *Malassezia* infections in dogs. Topical preparations containing miconazole, posaconazole, or clotrimazole are recommended for the treatment of both skin and ear infections. Oral drug formulations of itraconazole, at a dose of 5 mg/kg and administered once daily or pulse-ma-naged for at least 3 weeks, have been indicated for severe *Malassezia*-related disorders [10,11]. Given the increased resistance of *M. pachydermatis* to azoles, there is growing interest in both in vitro antifungal susceptibility testing and in the possibility of combination therapy against *M. pachydermatis* infections [12]. To improve the activity of less effective antifungals, some authors recommend combining different antifungal agents with each other or with chlorhexidine [13]. Using a checkerboard test, a synergistic effect was observed for combinations of itraconazole or ketoconazole with nystatin or caspofungin, clotrimazole with terbinafine or caspofungin, and miconazole with caspofungin [12]. Effective treatment of Basset hounds suffering from *M. pachydermatis*-associated seborrheic dermatitis with miconazole–chlorhexidine shampoo, resulting in a significant reduction in the yeast population not only on the skin surface but also in the oral cavity, has been reported [14]. Recent studies show good efficacy of phytotherapeutics, such as plant essential oils or their bioactive components, against *M. pachydermatis* or on its virulence factors [15,16,17,18]. However, there are still few studies evaluating the combination of antifungals with plant essential oil components against *M. pachydermatis.* For this reason, the aim of the present in vitro study was to evaluate the effect of combining selected azole antifungals with some plant essential oil components and to determine whether the combination of less effective antifungals with EO components showing high antifungal activity improves their activity.

## 2. Materials and Methods

### 2.1. Samples of Malassezia Pachydermatis

A total of 21 clinical isolates of *Malassezia pachydermatis* were included in the experiment. Swabs from the external ear canal of dogs diagnosed with *Malassezia* otitis were provided by the Small Animal Clinic (University of Veterinary Medicine and Pharmacy in Košice, Slovakia). The samples came from dogs of different ages (1–12 years old); breeds (Labrador Retriever—7 dogs; Hungarian Vizsla—4 dogs; Maltese Pinch—2 dogs; Cocker Spaniel—2 dogs; Yorkshire terrier—2 dogs; Cavalier King Charles Spaniel—1 dog; Newfoundland dog—1 dog; Mops—1 dog; Alpine Dachsbracke—1 dog); and genders (male—11; female—10). The *M. pachydermatis* strains used were identified and confirmed based on their phenotypic (macroscopic and microscopic) and genotypic characteristics (PCR-RFLP) [19,20,21]. Until use, isolates were stored at −80 °C in a freezing medium (100 μL of 60% glycerol and 300 μL of a medium—glucose, 4 g; tryptophan, 1 g; yeast extract, 0.5 g per 100 mL). Prior to use, the strains were revived twice on SAOT (Sabouraud’s dextrose agar—SDA—HiMedia Laboratories Pvt. Ltd., Mumbai, India, supplemented with gly- cerol—2 mL, Tween 80—2 mL, Tween 40—5 mL, and olive oil—5 mL per litre) and incubated at 35 °C for 96 h. The reference strain *M. pachydermatis* CBS 1879 (Centraalbureau voor Schimmelcultures, Utrecht, The Netherlands), derived from the ear of a dog with otitis externa, was also used for testing.

### 2.2. Determination of Minimum Inhibitory Concentration (MIC) of Tested Azole Antifungals and Plant Essential Oil Components

The efficacy of selected azole antifungals, posaconazole (POS); clotrimazole (CLO); miconazole (MCZ); itraconazole (ITR) (Sigma Aldrich, St. Louis, MO, USA); and plant essential oil (EO) components, eugenol, geraniol, terpinene-4-ol, and limonene (Sigma Aldrich, St. Louis, MO, USA), against a *M. pachydermatis* strain was determined by broth microdilution standard methods, M27-A3 [22], with certain modifications. SBOT (Sabouraud’s broth medium—HiMedia Laboratories Pvt. Ltd., Mumbai, India—supplemented with the same substances as SAOT) was used as the growth medium, and the final yeast inoculum reached 10^4^ CFU/mL. The test was performed in 96-well microtitre plates with a U-shaped bottom. The tested concentrations of azoles were prepared directly in the microtitre plate in wells 1–10 by two-fold dilution in the range from 32 to 0.0625 μg/mL. In the case of plant EO components, a stock 5% solution (50,000 μg/mL) was prepared by dissolving a 100% concentration of the EO component in 40% DMSO and consequently with SBOT so that the DMSO concentration reached 2%. The stock solution of each tested EO component was used to prepare the concentrations set up by binary dilution directly in microtitre plates (wells 1–10) ranging from 5 to 0.01% (50,000–100 μg/mL). After adding the *M. pachydermatis* inoculum suspension into the well of microtitre plates, the concentrations were halved in the range of 16 to 0.0313 μg/mL for antifungals and from 2.5 to 0.005% (25,000–50 μg/mL) for the EO components. Well 11, considered a negative control, contained only SBOT, and well 12, which served as a positive control, contained the inoculated medium. The microtitre plates were incubated at 35 °C for 72 h and 96 h, and then, the minimal inhibitory concentration (MIC) was read. For a better evaluation of the MIC end-points, 10 μL of 0.1% resazurin (sterilised through 0.22 μm filter before use) was added into each well of the microtitre plates six hours before reading the results. Inhibition of yeast growth was determined when the MIC prevented a change from blue (no yeast growth) to orange–pink (yeast growth) [23].

### 2.3. An Evaluation of the Efficacy of the Combination of Antifungal Agents and Plant Essential Oils Components

The checkerboard method was used to determine the interaction of selected antifungals and plant EO components.

Clotrimazole and miconazole, the azole antifungals that showed the lowest antifungal activity, were combined with eugenol, the plant EO component with the highest efficacy. Each tested agent was prepared at four-fold-higher concentrations based on their MIC_90_. When testing the combination of the azole antifungal with eugenol, the concentration of both combined agents halved once, and after the addition of yeast inoculum, the concentration was halved again. Thus, the final concentrations tested ranged from 16 to 0.0625 μg/mL for clotrimazole, from 2 to 0.008 μg/mL for miconazole, and from 0.16 to 0.0025% (1600–25 μg/mL) for eugenol. Clotrimazole or miconazole was added vertically in decreasing concentrations to microtitre plate wells 1–9 (within rows A–H) at a volume of 50 μL, whilst 50 μL of eugenol was added horizontally at decreasing concentrations in rows A–G (within wells 1–10). In this way, all concentrations of the tested azole were combined with each concentration of eugenol and vice versa. Inoculum of *M. pachydermatis* (100 μL) was applied in columns 1–10 and in column 12 (positive control containing 100 μL of inoculum and 100 μL of SBOT). Well 11 (negative control containing 200 μL of SBOT) served as a sterility control. One plate was used to test only one strain of *M. pachydermatis* and combinations of clotrimazole or miconazole with eugenol. The microtitre plates were incubated at 35 °C for 72 h or 96 h. Subsequently, the minimum inhibitory concentration of the combinations (MICCs) was assessed, which was used for the calculation of the fractionated inhibitory concentration index (FICI), according to the following formula:

(1) $$\mathrm{FICI}=\frac{\mathrm{MICC}}{\mathrm{MIC}}\mathrm{tested}\;\mathrm{azole}+\frac{\mathrm{MICC}}{\mathrm{MIC}}\mathrm{eugenol}$$

The MICC represents the minimum inhibitory concentration of the combined agents, and MIC is the minimum inhibitory concentration of the tested agents alone.

The results of the FICI values were interpreted as follows: synergistic (FICI ≤ 0.5), additive (0.5 < FICI ≤ 1.0), indifferent (1.0 < FICI ≤ 4.0), or antagonist (FICI > 4.0) [24].

Also, in this case, 10 μL of a 0.1% resazurin solution was added into the wells 6 h before reading the results.

### 2.4. Statistical Analysis

The experiments were performed twice for clinical strains and in triplicate for the reference strain, and average values were taken. The data are presented as averages ($\bar{x}$), standard deviations (SDs), modes, and medians. One-way ANOVA followed by Tukey’s multiple comparisons test was used to analyse the mean MICs of the selected antifungal agents with each other and also to compare the mean of FICI (efficiency of combinations) after 72 and 96 h of incubation (GraphPad Prism 8.0.1, San Diego, CA, USA). The level of statistical significance was set at *p* ˂ 0.05.

## 3. Results

The results of a statistical analysis of the MIC values of the tested azole antifungals are presented in Table 1. Out of the four agents tested, itraconazole showed the highest antifungal activity against clinical isolates of *M. pachydermatis* with an average MIC of 0.12 μg/mL and 0.14 μg/mL after 72 h and 96 h, respectively. Posaconazole, with a mean MIC of 0.12 μg/mL at 72 h and 0.19 μg/mL at 96 h, was another antifungal agent with high efficacy. Miconazole (a MIC mean of 1.71 μg/mL at 72 h and 2.33 μg/mL at 96 h) and clotrimazole (an average MIC of 7.62 μg/mL at 72 h and 7.24 μg/mL at 96 h) were the antifungals exhibiting the lowest activity. Statistically significant differences (*p* ˃ 0.05) were found when comparing the tested antifungals with each other but not when comparing the individual tested agents at 72 and 96 h.

Table 2 demonstrates the results of a statistical analysis of the MICs of the tested plant essential oil components. *M. pachydermatis* clinical isolates showed the highest susceptibility to eugenol (an average MIC of 378.57 μg/mL at 72 h and 1180 μg/mL at 92 h), followed by terpinene-4-ol (an average MIC of 428.57 μg/mL at 72 h and 1242.86 μg/mL at 92 h), and geraniol (an average MIC of 495.24 μg/mL at 72 h and 2082.38 μg/mL at 92 h). The significantly lowest inhibitory activity (*p* < 0.05) against *M. pachydermatis* strains was found for limonene, with an average MIC of 13,988.10 μg/mL at 72 h and 27,380.95 μg/mL at 92 h compared to other tested EO components or between 72 h and 96 h for limonene alone. The same order of antifungal activity for the tested plant EO components was also observed for the *M. pachydermatis* CBS 1879 strain.

When evaluating the results of the checkerboard assay, a decrease was noted when comparing the average MIC of clotrimazole and eugenol at 72 h and 96 h, tested alone, with average MICC values of the tested agents in combination (Table 3). However, no statistically significant differences were found, except when comparing the MIC of eugenol at 72 h (378.57 μg.mL^−1^) with MIC at 96 h (1180 μg.mL^−1^) and MIC with the MICC of eugenol at 96 h (1180 μg.mL^−1^ and 119.05 μg.mL^−1^). Similarly, significant differences were noticed between the MIC and MICC at 96 h for eugenol (800 μg.mL^−1^ and 50 μg.mL^−1^) in the *M. pachydermatis* CBS 1879 strain. Regarding the evaluation of FICI values in the clinical isolates, a significant decrease (*p* ˂ 0.05) was observed by comparing the mean FICI values at 72 (1.43) and 96 h (0.70). Although a decrease in FICI values was also recorded in the reference strain at 96 h (1.06) compared to 72 h (2.0), no statistically significant difference was found.

As for the combination of miconazole and eugenol, a decrease in MICCs was achieved at 72 and 96 h compared to the MICs of the tested agents alone (Table 4). Even in this case, a significant decline (*p* ˂ 0.05) was demonstrated when comparing the MICC of eugenol at 96 h (1180 μg.mL^−1^ and 147.62 μg.mL^−1^) in the clinical isolates, as well as in reference strain (800 μg.mL^−1^ and 50 μg.mL^−1^). In addition, a significant decrease in the mean FICI value was recorded in the isolates at 96 h (0.45) compared to the data at 72 h (1.30). However, in the reference strain, no statistically significant difference was observed when comparing FICIs at 72 (1.67) and 96 h (0.31).

Table 5 evaluates the effects of the combination of clotrimazole or miconazole with eugenol. As for the combination of clotrimazole with eugenol, after 72 h, a synergistic effect was noted in 3 isolates (14.19%), additive activity in 5 isolates (23.81%), indifferent efficacy in 11 isolates (52.38%), and an antagonistic effect in 2 isolates (9.52%). After 96 h, synergistic activity was detected in 3 isolates (14.29%), an additive effect was exhibited in 14 isolates (66.67%), and an indifferent effect was recorded in 4 strains (19.04%).

Regarding the combination of miconazole and eugenol, after 72 h, the effect was as follows: synergistic for 3 strain (14.29%), additive for 11 strains (52.38%), and indifferent for 7 isolates (33.33%). After 96 h, 12 isolates showed a synergistic effect, and 9 strains (42.86%) an additive effect.

## 4. Discussion

Successful treatment of *M. pachydermatis*-associated infections in dogs is based on the susceptibility of the yeast to chosen antifungals. Preparations based on azole antifungals are frequently used in clinical practice. Azoles exert their antifungal effect by the inhibition of CYP-dependent 14α-demethylase, an enzyme which is necessary for the conversion of lanosterol to ergosterol. Ergosterol, a major sterol found on the membrane of fungal pathogens, is important for the stability of the fungal cell membrane, and the inhibition of its biosynthesis compromises cell membrane integrity. Due to the accumulation of 14α-methyl sterol precursors, the properties and function of fungal cell membranes are altered, resulting in the obstruction of cell growth and division [25,26].

The first step of this study was to determine the susceptibility of *M. pachydermatis* against four azole antifungals. As the results show, posaconazole and itraconazole achieved the best antifungal activity. The tested strains exhibited less susceptibility to clotrimazole and miconazole. Similar to our findings, Bourdeau et al. [27] also reported the highest antifungal efficacy for posaconazole (MIC_90_ of 1–2 μg/mL) and less activity for clotrimazole (MIC_90_ of 16–32 μg/mL) and miconazole (MIC_90_ ≥ 32 μg/mL). In another study, *M. pachydermatis* strains isolated from dogs suffering from canine otitis or dermatitis were shown to be susceptible to itraconazole in the MIC range of 0.031–1 μg/mL and to posaconazole from 0.125 to 4 μg/mL [28]. Several studies point to the low susceptibility of *M. pachydermatis* to various antifungals provided by mono-drug therapies, usually associated with their long-term use, which is required to treat chronic *Malassezia* overgrowth and as a consequence of antifungal resistance [29,30,31]. Some authors recommend combining common antifungal agents used in clinical practice or antifungals with plant extracts, as they might provide effective alternative treatments against *M. pachydermatis* due to their synergistic interactions [32,33,34]. Most natural products exert selectivity in binding to fungi. Their primary molecular targets include fungal cell membranes, cell walls, and numerous organelles, in addition to acting as preventive agents [35]. In a study by Schlemmer et al. [33], the highest synergy interactions (80%) were observed for combinations of carvacrol with nystatin, thymol with nystatin, and carvacrol with miconazole against *M. pachydermatis*. Since there are no MIC breakpoints to determine the susceptibility of *M. pachydermatis*, as in the case for *Candida* species, azoles that showed a higher MIC value, indicating lower efficacy, were chosen for the experiments in which they were combined with plant essential oil components exhibiting high antifungal efficacy. Our in vitro study looked at the combination of selected azoles with plant essential oil components. Out of the four plant essential oil components tested, eugenol was chosen for the combination with miconazole and clotrimazole, as it demonstrated the best antifungal efficacy (mean MIC of 37,857 μg/mL at 72 h and 1180 μg/mL at 96 h) when tested alone. Eugenol is an aromatic compound belonging to the group of monoterpenoid phenols. It is the most important component of clove oil (*Syzygium aromaticum*), which is primarily responsible for its characteristic aroma [36]. Aiemsaard et al. [37] noted the effectiveness of eugenol against planktonic cells of *M. pachydermatis*, with a minimum planktonic inhibitory concentration (MPIC_50_) of 0.156 mg/mL and a minimum planktonic fungicidal concentration (MPFC_50_) of 0.312 mg/mL; however, these concentrations are higher compared to the results in the present study. Eugenol also displayed the best inhibitory effect on the production of extracellular phospholipase, one of the important virulence factors in *M. pachydermatis* strains [18]. When combining eugenol with clotrimazole, a decrease in the mean of MICC of both clotrimazole and eugenol was found after 72 h (3.67 μg/mL and 145.24 μg/mL) as well as after 96 h (3.95 μg/mL and 119.05 μg/mL) with an FICI value of 1.43 at 72 h and 0.70 at 96 h. The combination of eugenol with miconazole also led to a decrease in the mean of MICCs for miconazole to 0.83 μg/mL after 72 h and 0.57 μg/mL after 96 h and for eugenol, to 157.14 μg/mL after 72 h and 147.62 μg/mL after 72 h, with an FICI value of 1.30 at 72 h and 0.48 at 96 h. The decrease in the FICI value after 96 h was due to an increase in the synergistic effect, especially in the combination of miconazole with eugenol (12 isolates 57.14%) and the additive effect in the combination of clotrimazole with eugenol (14 isolates, 66.67%). Recent research shows that eugenol is able to interact with the cell membrane of *C. albicans*, reducing ergosterol biosynthesis and causing visible changes in the ultrastructure and morphology of the cell surface [38,39]. Based on this knowledge, we assume that eugenol supported a better effect in combinations with chosen azoles, especially after longer exposure. In conclusion, eugenol appears to be a promising natural compound with antifungal potential when used alone or in combination with a-zoles against *M. pachydermatis* strains.

## 5. Conclusions

Eugenol appears to be effective against *Malassezia*-associated infections. The combination of eugenol with less potent azole antifungals could also be very helpful in comba-ting infections caused by drug-resistant strains. However, in order for eugenol to be used in clinical practice as a suitable medication, further research is needed, such as for determining the cytotoxicity of eugenol with long-term exposure.

## Figures and Tables

**Table 1:** Results of a statistical analysis of MIC (μg/mL) of tested azole antifungals.

Parameter	Posaconazole		Clotrimazole		Miconazole		Itraconazole	
	72 h	96 h	72 h	96 h	72 h	96 h	72 h	96 h
Minimum	0.0625	0.0625	4	4	1	1	0.0625	0.0625
Maximum	0.25	0.25	16	8	2	4	0.25	0.5
$\bar{x}$	0.12 ^a,e,f^	0.19 ^a,g,h^	7.62 ^b^	7.24 ^b^	1.71 ^c^	2.33 ^c^	0.12 ^d,e,g^	0.14 ^d,f,h^
SD	0.04	0.07	3.32	1.61	0.46	0.86	0.06	0.13
Mode	0.125	0.25	8	8	2	2	0.125	0.125
Median	0.125	0.25	8	8	2	2	0.125	0.125
MIC_50_	0.125	0.25	8	8	2	2	0.125	0.125
MIC_90_	0.125	0.25	8	8	2	4	0.25	0.25
*Malassezia pachydermatis* CBS 1879								
Minimum	0.0625	0.25	4	4	1	4	0.125	0.5
Maximum	0.125	0.25	8	4	2	4	0.125	0.5
$\bar{x}$	0.10 ^i,j,k,l^	0.25 ^i,m,n,o^	6.67	4 ^p,q^	1.67 ^j,m,p,r,s,t^	4 ^q,r^	0.125 ^k,n,s,u^	0.5 ^l,o,t,u^
SD	0.04	0	2.31	0	0.58	0	0	0
Mode	0.125	0.25	8	4	2	4	0.125	0.5
Median	0.125	0.25	8	4	2	4	0.125	0.5

$\bar{x}$—average of MIC, MIC_50_, and MIC_90_—minimum inhibitory concentration at which 50% and 90% of the strains were inhibited; ^a–u^—MIC values with the same superscript letter are not statistically significantly different (*p* > 0.05).

**Table 2:** Results of a statistical analysis of MIC (μg/mL) of tested plant essential oil components.

Parameter	Eugenol		Geraniol		Terpinen-4-ol		Limonen	
	72 h	96 h	72 h	96 h	72 h	96 h	72 h	96 h
Minimum	50	200	200	400	200	400	6250	25,000
Maximum	1600	6250	800	25,000	800	12,500	25,000	50,000
$\bar{x}$	378.57 ^a,b^	1180 ^c,d^	495.24 ^e,f^	2082.38 ^g,h^	428.57 ^i,j^	1242.86 ^k,l^	13,988.10 ^a,c,e,g,i,k,m^	27,380.95 ^b,d,f,h,j,l,m^
SD	423.25	1304.97	257.83	5278.89	202.84	2595.10	4802.65	7519.82
Mode	200	800	800	800	400	800	12,500	25,000
Median	200	800	400	800	400	800	12,500	25,000
MIC_50_	200	800	400	800	400	800	12,500	25,000
MIC_90_	400	6250	800	1600	800	800	25,000	25,000
*Malassezia pachydermatis* CBS 1879								
Minimum	50	800	200	25,000	100	5000	12,500	50,000
Maximum	100	800	200	25,000	200	25,000	12,500	50,000
$\bar{x}$	83.33 ^n,o^	800 ^p,q,r^	200 ^n,p,s,t^	25,000	133.33 ^o,q,r,s,u^	11,666.67 ^r,t,u,v^	12,500 ^s,v^	50,000
SD	28.86	0	0	0	57.74	11,547.01	0	0
Mode	100	800	200	25,000	100	5000	12,500	50,000
Median	100	800	200	25,000	100	5000	12,500	50,000

$\bar{x}$—average of MIC, MIC_50_, and MIC_90_—minimum inhibitory concentration at which 50% and 90% of the strains were inhibited; ^a–m^—MIC values for isolates with the same superscript letter are statistically significantly different (*p* ˂ 0.05); ^n–v^—MIC values for reference strain with the same superscript letter are not statistically significantly different (*p* ˂ 0.05).

**Table 3:** Results of an evaluation of MIC (μg/mL) and MICC (μg/mL) of the combination of clotrimazole and eugenol and FICI values.

Parameter	Clotrimazole		Eugenol		FICI	Clotrimazole		Eugenol		FICI
	MIC/72 h	MICC/72 h	MIC/72 h	MICC/72 h		MIC/96 h	MICC/96 h	MIC/96 h	MICC/96 h	
Minimum	**4**	1	50	50	0.31	4	1	200	50	0.38
Maximum	16	8	1600	400	4.50	8	8	6250	200	1.25
$\bar{x}$	7.62	3.67	378.57 ^a^	145.24	1.43 *	7.24	3.95	1180 ^a,b^	119.05 ^b^	0.70 *
SD	3.32	1.77	423.25	98.62	1.13	1.61	1.63	1304.97	55.85	0.25
Mode	8	4	200	100	1.50	8	4	800	100	0.75
Median	8	4	200	100	1.25	8	4	800	100	0,63
MIC_50_	8	4	200	100	-	8	4	800	100	-
MIC_90_	8	4	400	200	-	8	4	6250	200	-
*Malassezia pachydermatis* CBS 1879										
Minimum	4	4	50	100	1.50	4	4	800	50	1.06
Maximum	8	4	100	100	3	4	4	800	50	1.06
$\bar{x}$	6.67	4	83.33 ^c^	100 ^d^	2	4	4	800 ^c,e^	50 ^d,e^	1.06
SD	2.31	0	28.87	0	0.86	0	0	0	0	0
Mode	8	4	100	100	1.50	4	4	800	50	1.06
Median	8	4	100	100	1.50	4	4	800	50	1.06

$\bar{x}$—average of MIC or MICC, MIC_50_, and MIC_90_—minimum inhibitory concentration at which 50% and 90% of the strains were inhibited; ^a–e,^ *—MIC, MICC, and FICI values with the same superscript are statistically significantly different (*p* < 0.05).

**Table 4:** Results of a statistical analysis of MIC (μg/mL) and MICC (μg/mL) of the combination of miconazole and eugenol.

Parameter	Miconazole		Eugenol		FICI	Miconazole		Eugenol		FICI
	MIC/72 h	MICC/72 h	MIC/72 h	MICC/72 h		MIC/96 h	MICC/96 h	MIC/96 h	MICC/96 h	
Minimum	1	0.5	50	100	0.38	1	0.25	200	50	0.16
Maximum	2	1	1600	200	3.00	4	1	6250	400	0.75
$\bar{x}$	1.71	0.83	378.57 ^a^	157.14	1.30 *	2.33	0.57	1180 ^a,b^	147.62 ^b^	0.45 *
SD	0.46	0.24	423.25	50.71	0.80	0.86	0.30	1304.97	104.25	0.22
Mode	2	1	200	200	1	2	0.5	800	200	0.38
Median	2	1	200	200	1	2	0.5	800	100	0.38
MIC_50_	2	1	200	100	-	2	0.5	800	100	-
MIC_90_	2	1	400	200	-	4	0.5	6250	200	-
*Malassezia pachydermatis* CBS 1879										
Minimum	1	0.5	50	100	1.25	4	1	800	50	0.31
Maximum	2	0.5	100	100	2.50	4	1	800	50	0.31
$\bar{x}$	1.67	0.5	83.33 ^c^	100 ^d^	1.67	4	1	800 ^c,e^	50 ^d,e^	0.31
SD	0.58	0	28.87	0	0.72	0	0	0	0	0
Mode	2	0.5	100	100	1.25	4	1	800	50	0.31
Median	2	0.5	100	100	1.25	4	1	800	50	0.31

$\bar{x}$—average of MIC or MICC, MIC_50_, and MIC_90_—minimum inhibitory concentration at which 50% and 90% of the strains were inhibited; ^a–e,^ *—MIC, MICC, and FICI values with the same superscript are statistically significantly different (*p* < 0.05).

**Table 5:** Results of an evaluation of the effect of the combination of azoles with eugenol.

Effect	Clotrimazole × Eugenol		Miconazole × Eugenol	
	72 h	96 h	72 h	96 h
Synergistic (n/%)	3/14.29	3/14.29	3/14.29	12/57.14
Additive (n/%)	5/23.81	14/66.67	11/52.38	9/42.86
Indifferent (n/%)	11/52.38	4/19.04	7/33.33	0
Antagonistic (n/%)	2/9.52	0	0	0
*Malassezia pachydermatis* CBS 1879				
Synergistic (n/%)	0	0	0	3/100
Additive (n/%)	0	3/100	2/66.67	0
Indifferent (n/%)	3/100	0	1/33.33	0
Antagonistic (n/%)	0	0	0	0

## Data Availability

The original contributions presented in the study are included in the article, further inquiries can be directed to the corresponding author.
